# An evaluation of the incorporation of psychological interventions into the care of patients with a diagnosis of emotionally unstable personality disorder following admission to the general adult inpatient setting

**DOI:** 10.1192/bjo.2021.859

**Published:** 2021-06-18

**Authors:** Declan Hyland, Charlie Daniels, Iulian Ionescu, Katie Goodier, Simon Graham

**Affiliations:** 1 Consultant Psychiatrist, Clock View Hospital, Liverpool, Mersey Care NHS Foundation Trust; 2 Core Trainee 2 in Psychiatry, Spring House, Liverpool, Mersey Care NHS Foundation Trust; 3 Higher Trainee in Medical Psychotherapy, Spring House, Liverpool, Mersey Care NHS Foundation Trust; Christina Houghton, Foundation Year 2 trainee, Clock View Hospital, Liverpool, Mersey Care NHS Foundation Trust; 4 Foundation Year 1 Trainee, Clock View Hospital, Liverpool, Mersey Care NHS Foundation Trust; 5 Consultant in Medical Psychotherapy, Spring House, Liverpool, Mersey Care NHS Foundation Trust

## Abstract

**Aims:**

To assess incorporation of and access to psychological therapies for patients with a diagnosis of emotionally unstable personality (EUPD) who were discharged from the inpatient wards at Clock View Hospital, an inpatient unit in Mersey Care NHS Foundation Trust.

**Method:**

A retrospective analysis of the electronic record of 50 patients discharged from Clock View Hospital between 1st of January 2020 and 1st of November 2020 was performed to assess whether patients were engaged with psychotherapy and whether they had an extended care plan in place.

25 patients with EUPD and no associated psychiatric comorbidities were included in the sample, as well as 25 patients with EUPD and associated psychiatric comorbidities.

**Result:**

Those EUPD patients with no psychiatric comorbidities were more likely to be under the care of the Liverpool Personality Disorder (PD) Hub compared to those with psychiatric comorbidities (12 vs seven patients). Of the 19 patients under the PD Hub, 11 had a Case Manager, four were engaged with the PD Hub's day services / safe service and one with a PD Hub readiness group. Six of the 50 patients had a documented refusal to engage with the PD Hub.

Only 27 of the patients had either received psychological intervention, were on a waiting list, or had a referral in place. 16% of patients refused a psychotherapy referral. Of the 20 patients who received psychological treatment, eight completed a form of psychotherapy (cognitive analytic therapy, dialectical behaviour therapy, cognitive behavioural therapy, eye movement desensitisation and reprocessing) and 12 psychological intervention (either structured case management, psychoeducation or emotional coping skills).

Only 28 of 50 patients had an extended care plan and 28 had a collaborative risk management plan in place.

**Conclusion:**

There was no obvious correlation between previous completion of psychological therapy and degree of polypharmacy. Median admission time was reduced for patients under the PD Hub (six vs 14 days). This was also reduced for patients who accessed psychotherapy or psychotherapeutic interventions (nine vs 10 days).

This audit coincided with the COVID-19 pandemic and subsequent reduced access to the PD Hub and psychotherapy service. There is a need to consider barriers to EUPD patients receiving psychotherapy.

EUPD patients may have numerous hospital admissions and frequently present in crisis. Given the iatrogenic harm from prolonged hospital admission, there is a need to consider incorporating a collaborative extended care plan and risk management plan as part of discharge planning, following admission to hospital.

